# Supplementary material for: Marine Bacterial Polysaccharide EPS11 Inhibits Cancer Cell Growth and Metastasis via Blocking Cell Adhesion and Attenuating Filiform Structure Formation
Source: Mar Drugs. 2019 Jan 11;17(1):50. doi: 10.3390/md17010050 (PMC6357516; doi:10.3390/md17010050)
Supplement: Supplementary file 1 [file marinedrugs-17-00050-s001.pdf]

Supplementary information

Marine Bacterial Polysaccharide EPS11 Inhibits Cancer Cell Growth and Metastasis via Blocking Cell Adhesion and Attenuating Filiform Structures Formation

Ju wang[1, 2, 3], Ge Liu[1, 2,4], Weiping Ma[5], Zhongxia Lu[5], and Chaomin Sun[1, 2,4*]

[1]CAS Key Laboratory of Experimental Marine Biology, Institute of Oceanology, Chinese Academy of Sciences, Qingdao 266071, China

[2]Laboratory for Marine Biology and Biotechnology, Qingdao National Laboratory for Marine Science and Technology, Qingdao 266071, China

[3]University of Chinese Academy of Sciences, Beijing 100049, China

[4]Center for Ocean Mega-Science, Chinese Academy of Sciences, Qingdao 266071, China

[5]School of Medicine and Pharmacy, Ocean University of China, Qingdao 266003, China

[*]Corresponding: Chaomin Sun, sunchaomin@qdio.ac.cn; Tel.: +86-532-82898857; fax: +86-532-82898648.

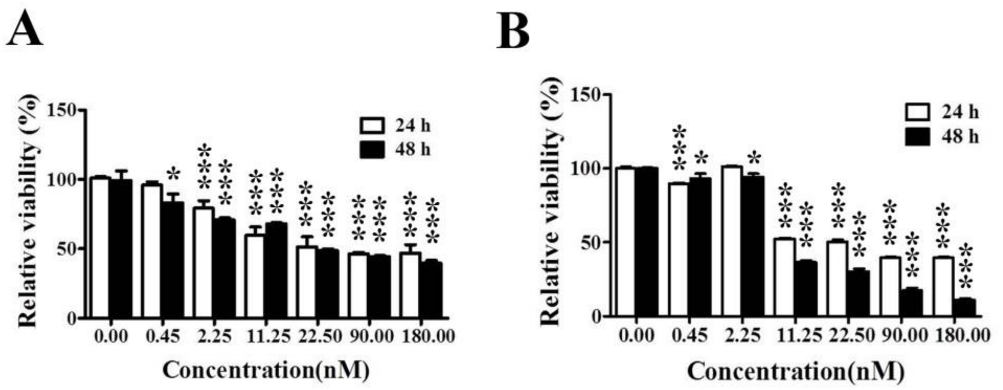

Figure S1. Cytotoxic effects of EPS11 on liver cancer cell lines 7402 (**A**) and HepG2 (**B**). Cells were seeded in 96-well plate overnight, and treated with different concentrations of EPS11 for 24 hours and 48 hours, respectively. The cell viability was analyzed by MTT assay. Data were presented as means ± SD of three independent experiments (n = 3). * $p < 0.05$, ** $p < 0.01$, *** $p < 0.001$.

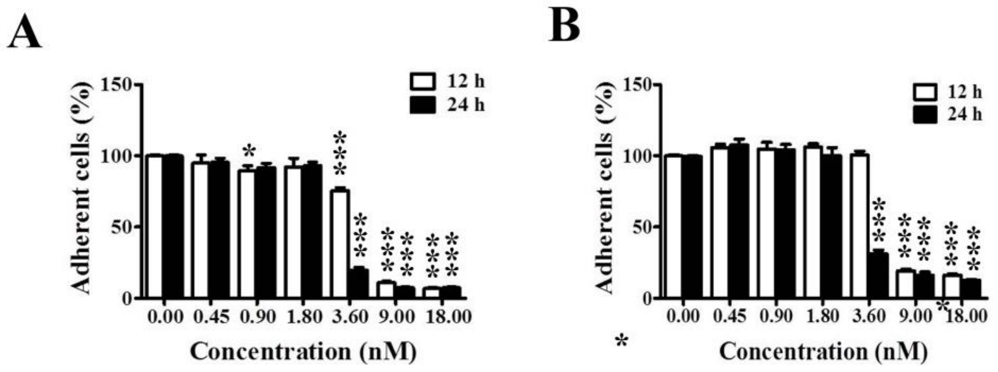

Figure S2. Quantification assay of cell adhesion in liver cancer cell lines 7402 (**A**) and HepG2 (**B**) after treatment with different concentrations of EPS11 for 12 hours and 24 hours, respectively. The data were presented as means ± SD of three observation fields in one representative experiment chosen from three independent experiments. *$p < 0.05$, **$p < 0.01$, ***$p < 0.001$.
